# Incidentally discovered huge retroperitoneal mucinous cystadenoma with successful laparoscopic management: Case report

**DOI:** 10.1016/j.ijscr.2019.07.023

**Published:** 2019-07-19

**Authors:** Mohammed S. Foula, Abdullah S. AlQattan, Ahmed M. AlQurashi, Hassan M. AlShaqaq, M. Khalid Mirza Gari

**Affiliations:** a Imam Abdulrahman Bin Faisal University, King Fahad University Hospital, Department of Surgery, Saudi Arabia; b Imam Abdulrahman Bin Faisal University, Saudi Arabia

**Keywords:** Retroperitoneal cyst, Mucinous cystadenoma, Laparoscopy, Literature review

## Abstract

**Introduction:**

Retroperitoneal cystic lesions are uncommon heterogeneous clinical entities with no definite incidence. Their clinical presentations are different and their diagnosis is challenging. The management necessitates complete surgical excision, usually via laparotomy. Recently, laparoscopic approach is being increasingly used.

**Case presentation:**

A 29-year-old female patient was referred for an incidentally-discovered huge retroperitoneal cyst. Imaging studies revealed a retroperitoneal cyst, measuring 13 * 11 cm. Diagnostic laparoscopy showed a retroperitoneal cyst displacing the small bowel and the right colon to the left side. The peritoneal covering was dissected from the cyst with caution not to cause cyst rupture. The cyst was removed partially using Endobag, then aspiration of its content to facilitate its delivery. The patient had a smooth uneventful postoperative course.

**Discussion:**

The retroperitoneal space is large, expandable space which enables retroperitoneal cystic lesions to grow asymptomatic. CT scan remains the best imaging modality. Aspiration of its content is not routinely done as its sensitivity and specificity has been reported low. Moreover, it carries the risk of leakage of the cyst content into the peritoneal space. Open surgical complete excision is the traditional management and remains of choice. However, laparoscopic management can be tried with caution not to cause content spillage. Intraoperatively, controlled aspiration of the cyst helps in its retrieval.

**Conclusion:**

Primary retoperitoneal mucinous cystadenoma is a rare clinical entity that is usually incidentally discovered. Laparoscopic excision is safe and feasible if done by an expert laparoscopic surgeon. Care should always be taken not to cause spillage of its content.

## Highlights

• Retroperitoneal cystic lesions are uncommon heterogeneous clinical entities with no clear pathogenesis.
• Their clinical presentations are different with challenging diagnosis. Many cases may be discovered incidentally.
• Care must be taken during the operation not to cause spillage of its content.
• Laparoscopic excision is technically challenging but can be done safely by experienced laparoscopic surgeon.

## 1 Introduction

Retroperitoneal cystic lesions are uncommon heterogeneous clinical entities with no definite incidence nor clear pathogenesis. Particularly, primary retroperitoneal cyst (PRPC) is considered as a rare type of these lesions. Two factors contribute to this rarity: absence of the epithelial lining of the retroperitoneum, as well, its large space extending form the diaphragm to the pelvis which allows such cysts to enlarge significantly without any specific symptoms. Their clinical presentations are different and their diagnosis is challenging. Patients usually present with nonspecific vague abdominal symptoms, obstructive symptoms may be evident in large masses [1,2].

Retroperitoneal cystic lesions can be classified into neoplastic and non-neoplastic subgroups. Neoplastic primary retroperitoneal cysts include cystic lymphangioma, mucinous cystadenoma, cystic teratoma, cystic mesothelioma, mullerian cyst, epidermoid cyst, bronchogenic cyst, cystic change in solid neoplasms, pseudomyxoma retroperitonei, and perianal mucinous carcinoma. The management necessitates complete surgical excision, usually via laparotomy. Recently, laparoscopic approach is being increasingly used, typically, with aspiration of the cyst to facilitate its dissection and to avoid spillage of its content. Other modalities were described in the literature as SAND balloon [2,3].

We report a case of young female with incidental finding of huge retroperitoneal cyst which was managed laparoscopically. This work is reported in line with SCARE criteria [4].

## 2 Case presentation

**Fig. 1:**
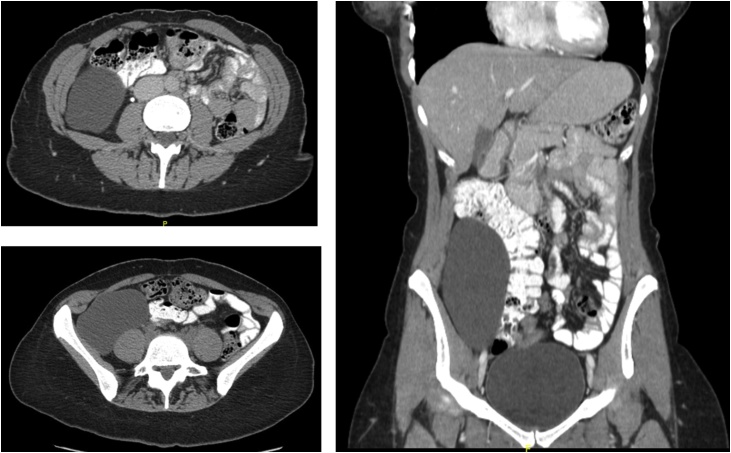
Contrast-enhanced CT abdomen showing a huge uni-loculated mass in the right side lying over the paravertebral muscles and displacing the right colon and whole bowel to the left side. The mass is extending in right para-colic gutter from hepatic flexure down to the uterus.

A 29-year-old female patient, who was not known to have any medical illnesses with previous surgical history of a cesarean section, was referred to the surgical clinic after an incidental finding of a huge cyst in her gynecological ultrasonography. Interestingly, there was a cystic oval-shaped lesion in the right hypochondrium extending caudally to right iliac fossa. Contrast-enhanced computed tomography revealed a huge retroperitoneal cyst, uni-loculated, measuring 13 * 11 cm, laterally to the right colon. All requested blood works were unremarkable (Fig. 1).

The patient was scheduled for elective diagnostic laparoscopy for excision of the retroperitoneal cyst with possible conversion to laparotomy. She was placed in supine, Trendlenberg position with adducted arms. Pneumoperitoneum was achieved by Veress needle in Palmer’s point. Insertion of 30 degree scope was done through an 11-mm Visi-port. Diagnostic laparoscopy confirmed presence of retroperitoneal cyst displacing the small bowel and the right colon to the left side and covered by a thin layer of peritneum. Two 5-mm ports were inserted in the left midclavicular lines, and suprapubic. Using combination of sharp dissection with Enseal® and blunt dissection, the peritoneal covering was dissected from the cyst with caution not to cause cyst rupture and consequent spillage of its contents. The cyst was removed partially using Endobag, then aspiration of its content outside the abdominal cavity to facilitate its delivery.

**Fig. 2:**
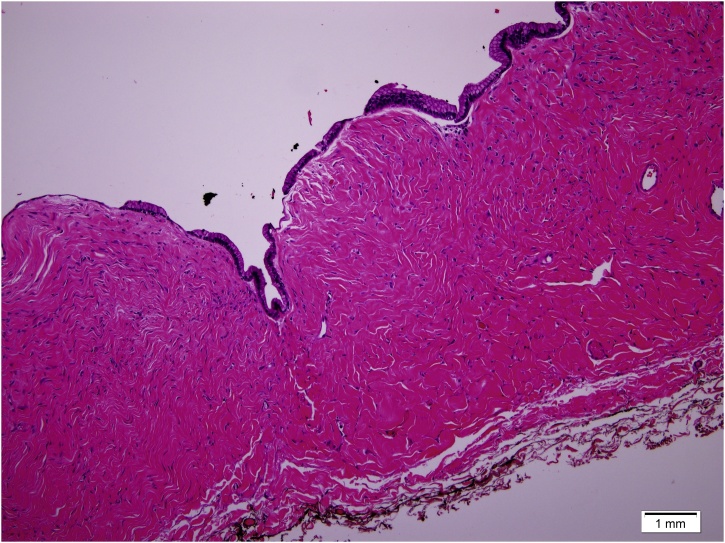
Histopathological examination showing a single layer of columnar non-ciliated epithelial cells, with basal nuclei and abundant intracellular pale mucinous fluid.

The patient had a smooth uneventful postoperative course. She was discharged home in a good condition on the second postoperative day. Histopathological examination exhibited a single layer of columnar non-ciliated epithelial cells, with basal nuclei and abundant intracellular pale mucinous fluid, which is consistent with primary retroperitoneal mucinous cystadenoma (Fig. 2). She was followed up in the surgical clinic regularly with no complaint. After six months, follow up CT excluded recurrence. She was planned for biannually clinic visit with annual CT scan.

## 3 Discussion

Primary retroperitoneal mucinous cystadenoma is a rare clinical entity with no absolute incidence reported in the literature. Pesapane et al reported 37 cases after performing a comprehensive literature review from 1980 until May 2017, using the search terms ‘primary’, ‘retroperitoneal’,’ mucinous ‘and ‘cystadenoma’ or ‘cystic adenoma’ in title, abstract and/or keywords through Medline, Scopus and Google Scholar databases [5]. We have found four more cases in English literature after conducting a literature review using the similar criteria to collect the reported cases after 2017 (Table 1). Moreover, the current case is the second case to be reported in Saudi Arabia after another case published in 2009 [6].

**Table 1:** Reported cases of primary retroperitoneal mucinous cystadenoma from 2017 up to date.

Year of publication	Study	Gender	Age at diagnosis	Tumor size (cm)	Surgical technique	Follow-up (months)	status
2017	Nardi et al. [7]	Female	50	17 × 15 × 13 cm	Laparoscopy	N/R	N/R
2018	Taga et al. [8]	Female	34	16 × 8 cm	Laparoscopy	12	NED
2018	Petrović [9]	Female	50	100 × 70 × 80 mm	Laparoscopy	N/A	N/A
2019	Koyama et al. [10]	Female	41	50 × 22 × 30 mm	Laparoscopy	N/R	N/R
Current case	Current case	Female	29	13 × 11 cm	Laparoscopy	6	NED

N/A: Not Available; NED: No evidence of disease; N/R: Not reported.

The retroperitoneal space is large, expandable space that is confined anteriorly by the parietal peritoneal covering, posteriorly by the psoas and quadratus lumborum muscles, superiorly by the diaphragm, and inferiorly by the pelvic muscles. Therefore, it enables retroperitoneal cystic lesions to grow asymptomatic for a long period of time before presenting with vague symptoms or pressure symptoms of adjacent organs [1].

As the retroperitoneal cysts lesions represent wide variety of lesions ranging from simple benign to malignant lesions with no specific clinical criteria, preoperative investigations should be done to determine the exact type. Unfortunately, diagnosis is not easily achievable. Use of serological investigations, ultrasonography, CT, and magnetic resonance imaging (MRI) helps, although, it cannot allow a confident diagnosis. Once diagnosed, it should be entirely excised because it carries the risk of rupture, infection or theoretical malignant degeneration.

CT scan remains the gold standard imaging modality that can detect and reveal some features including: location, size, wall thickness, enhancement pattern, regularity, invasion of the nearby organs or presence of calcifications, fat, myxoid stroma and/or necrosis. Nevertheless, MRI has a better yield, compared to CT, as it can show enhancing intra-lesion septae or nodules as well as more accurate localization and extension. However, imaging modalities cannot exclude the malignant potential of the retroperitoneal masses [1,2,11].

Aspiration of its content can reveal the epithelial cell type that may help in determining the histological subtype, however, it is not routinely done as its sensitivity and specificity has been reported low. Additionally, it carries the risk of leakage of the cyst content into the peritoneal space leading a serious pseudomyxoma peritonei or seeding of cells in case of malignancy [11,12].

Open surgical complete excision is the traditional management and remains of choice. However, with presence of expert laparoscopic surgeon, laparoscopic management can be tried with caution not to cause content spillage. Intraoperatively, controlled aspiration of the cyst helps in its retrieval. Some authors reported the use of the SAND balloon catheter, a specially designed catheter which was first described by Ikuma et al in 1998 for laparoscopic ovarian cystectomy [13]. Recurrence rate was reported in 25% in a case series, however, there is no accurate figures in the literature as cases are sporadically reported [1,3,12].

Postoperative histopathological examination is usually conclusive. Its typical findings are a cyst lined by a single layer of tall columnar non-ciliated epithelium with pale mucin-containing cytoplasm and surrounded by dense fibrous tissues with basal nuclei.

In our case, retroperitoneal cyst was discovered incidentally without any specific symptoms, and its criteria was delineated using CT. We preferred not to do aspiration cytology but to go directly for a trial of laparoscopic removal which was successful. Histopathological examination confirmed the diagnosis of primary retroperitoneal mucinous cystadenoma. As these lesions lack guidelines, we proposed to follow her with annual CT scan if remained symptomatic asymptomatic.

## 4 Conclusion

Primary retroperitoneal mucinous cystadenoma is a rare clinical entity that is usually incidentally discovered. Laparoscopic excision is safe and feasible if done by an expert laparoscopic surgeon. Care should always be taken not to cause spillage of its content.

## Sources of funding

No funds or sponsors.

## Ethical approval

Case reports are exempted from ethical approval according to local institutional policies.

## Consent

Written informed consent was obtained from the patient for publication of this Case report and any accompanying images. A copy of the written consent is available for review by the Editor-in-Chief of this journal on request.

## Author’s contribution

Dr. Mohammed S. Foula: main author, writing the paper, reviewing article.

Dr. Abdullah ALQattan: data collection, writing the paper.

Dr. Ahmed M AlQurashi, data collection.

Dr. Hassan M AlShaqaq: data collection, Writing the paper.

Dr. M Khalid Mirza Gari: supervisor, reviewing article, Treating Consultant.

## Registration of research studies

None.

## Guarantor

Mohammed S. Foula

## Provenance and peer review

Not commissioned, externally peer-reviewed.

## Declaration of Competing Interest

No conflict of interests.
